# Successful management of a tracheomalacia patient with active endotracheal bleeding due to intraoperative innominate artery injury: A case report

**DOI:** 10.1097/MD.0000000000030797

**Published:** 2022-09-30

**Authors:** Yoo Jung Park, Eunji Kim, Hong Soo Jung

**Affiliations:** a Department of Anesthesiology and Pain Medicine, St. Vincent’s Hospital, College of Medicine, The Catholic University of Korea, Seoul, Republic of Korea.

**Keywords:** endotracheal bleeding, tracheo-innominate artery fistula, tracheomalacia

## Abstract

Intraoperative innominate artery injury is life-threatening in tracheomalacia patients with prolonged tracheostomy. Anesthetic management is challenging in cases with massive hemorrhage into the endotracheal tube. We report a case in which we successfully managed a tracheomalacia patient with acute endotracheal bleeding due to innominate artery injury.

**Patient concerns:**

A 24-year-old patient with tracheomalacia was scheduled to undergo exploratory thoracotomy for the treatment of intermittent bleeding at the tracheostomy site. During exploration, sudden active bleeding due to innominate artery injury was observed in the endotracheal lumen.

**Diagnoses:**

The patient was diagnosed with tracheomalacia.

**Interventions:**

We immediately used the bronchoscope to place the tip of the endotracheal tube at the bleeding site and hyperinflated the cuff.

**Outcomes:**

The ballooned cuff compressed the active bleeding site, so no additional bleeding was detected by bronchoscopy, and no additional massive bleeding was observed in the operative field.

**Lessons:**

Immediate and appropriate overinflation of the endotracheal tube cuff by an anesthesiologist may provide improved surgical field visibility and time for critical surgical procedures in cases of massive hemorrhaging.

## 1. Introduction

Tracheomalacia refers to diffuse weakness of the tracheal wall due to the loss of the structural integrity of the tracheal cartilage.^[1]^ Tracheostomy and indwelling endotracheal tubes with cuffs are the most common causes of acquired tracheomalacia in adults.^[2]^ Because of the flaccidity of the tracheal wall, patients with tracheomalacia are susceptible to tracheal injury caused by the distal end of the tracheostomy tube. This leads to erosion of the tracheal wall and movement of the tracheostomy tube into the major vessels, such as the innominate artery.^[3]^ Tracheo-innominate artery fistula (TIF) is a life-threatening complication of prolonged tracheostomy; this is especially true in patients with tracheomalacia. Although several cases of TIF after tracheostomy have been reported,^[4–6]^ to our knowledge, intraoperative innominate artery injury in a patient with tracheomalacia is rare.

We report a case in which we successfully managed a tracheomalacia patient with acute endotracheal bleeding by prompt overinflation of the endotracheal tube cuff during surgery.

## 2. Case report

A 24-year-old female with tracheobronchomalacia was scheduled to undergo exploratory thoracotomy for the treatment of intermittent bleeding at the tracheostomy site. The patient weighed 48 kg and was 160 cm in height. She had been in a pedestrian traffic accident in 2006 and underwent emergency craniectomy and ventriculoperitoneal shunting. Afterward, she suffered from meningitis and panperitonitis numerous times. She was treated for these conditions in a local hospital ICU and underwent tracheostomy. She had no other remarkable medical history.

**Figure 1.**
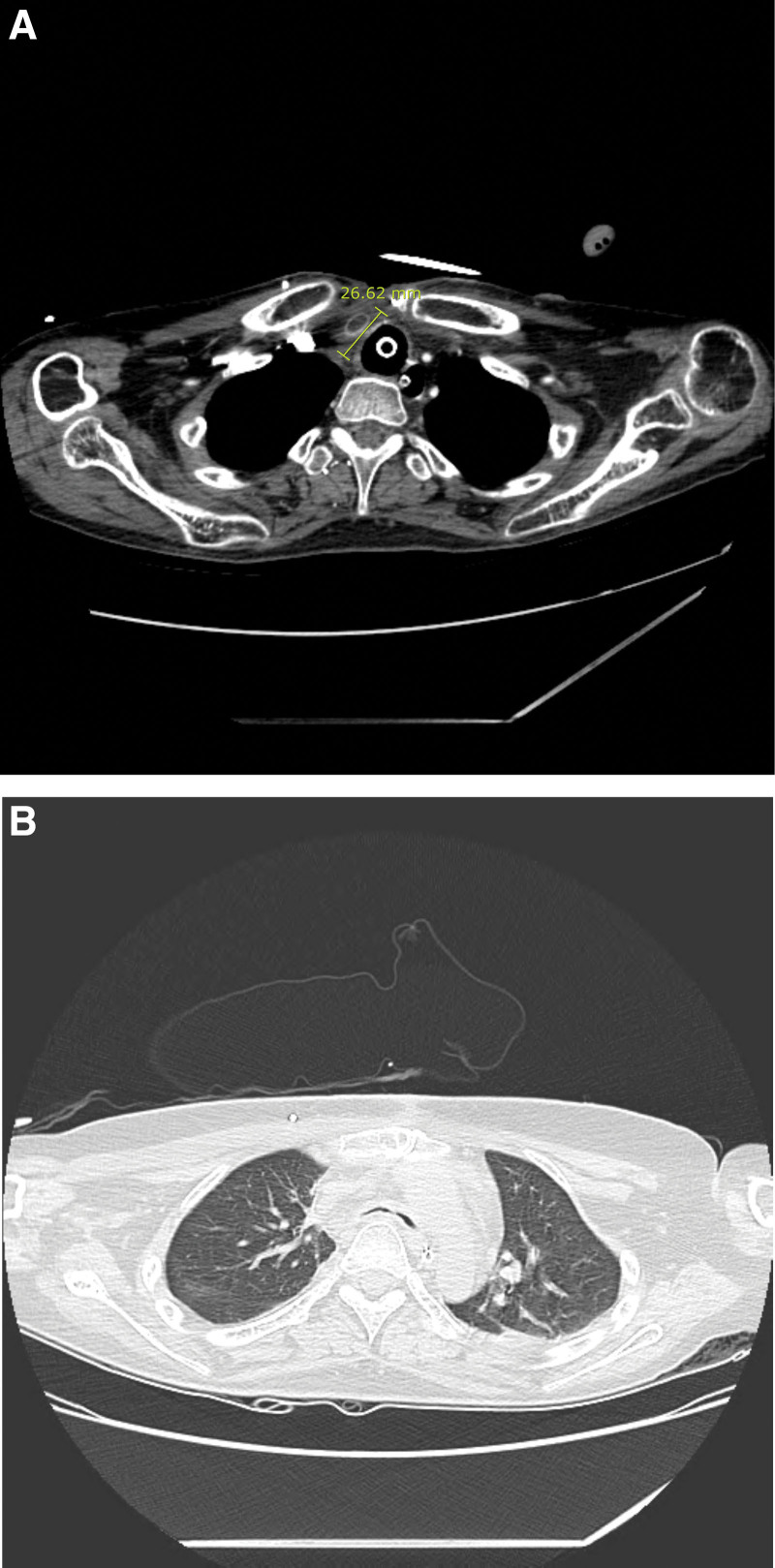
(A and B) Preoperative chest CT revealing diffuse tracheal wall thickening and advanced luminal narrowing of the trachea.

Preoperative chest CT revealed the progression of extensive tracheobronchitis with profuse endotracheal/bronchial secretions as well as diffuse wall thickening and advanced luminal narrowing of the trachea, implying tracheomalacia (Fig. 1A and B). The preoperative laboratory results were within normal limits, except those for hemoglobin (8.6 g/dL), INR (2.26), and activated partial thromboplastin time (121.0). A left anterior fascicular block was seen on her electrocardiogram. The patient and her family had no notable history related to anesthesia or drugs.

On arrival to the operating room, the patient’s vital signs were as follows: blood pressure (BP), 85/40 mm Hg; heart rate (HR), 108 beats/minute; respiratory rate, 22 breaths/minute; and body temperature, 36.0 °C. The patient’s oxygen saturation as measured by pulse oximeter while receiving air was 80%, which was partially corrected to 100% with Ambu bagging and blood suction. Etomidate (10 mg) was administered to induce anesthesia. After muscle relaxation with 40 mg IV rocuronium, the tracheostomy port was removed, and tracheal intubation was performed with a reinforced tube (inner diameter, 7.0 mm). Sevoflurane 1.5 vol%:O_2_:air was administered to maintain anesthesia. After anesthetic induction, a BP of 141/93 mm Hg and HR of 114 beats/minute were noted. An arterial catheter was inserted into her radial artery.

To find the bleeding site, exploration from the stoma site to the sternal notch was performed. During the exploration, the surgeon asked for a slight withdrawal of the endotracheal tube, so we deflated the ballooned cuff and pulled out the endotracheal tube approximately 1 cm with a visible bronchoscope. At that moment, we could see, by bronchoscopy, active bleeding in the endotracheal lumen. A BP of 74/45 mm Hg and HR of 130 beats/minute were noted, and transfusion was started. We pushed the endotracheal tube approximately 3 cm with blood suction by bronchoscopy and overinflated the cuff. The ballooned cuff compressed the active bleeding site. Thus, no additional bleeding was detected by bronchoscopy, and no additional massive bleeding was observed in the operative field. Vital signs improved to 102/62 mm Hg BP and 109 beats/minute HR, and we notified the surgeon accordingly. They identified innominate artery injury and ligated the bleeding vessels after sternotomy. The surgeon located the tracheal rupture site in the lower portion, covered the pericardial flap and finished the operation with a vacuum dressing. The patient was transfused with four units of packed red blood cells to counter further blood loss. Hemoglobin, measured by a blood gas analyzer, was 9.9 g/dL at the end of surgery.

This study was approved by the Institutional Review Board of our University Hospital. Written informed consent was obtained from the patient for the publication of this case report.

## 3. Discussion

Tracheomalacia is defined as the collapse of more than 50% of the cross-sectional area of the trachea during expiration or coughing.^[1,7]^ Tracheomalacia is classified into congenital and acquired forms. Congenital tracheomalacia is caused by the immaturity of tracheobronchial cartilage, which results in a loss of the structural integrity of the entire trachea. Congenital tracheomalacia has been associated with other pediatric abnormalities, such as tracheoesophageal fistula and congenital heart disease.^[8–10]^ Acquired tracheomalacia results from various potential causes that can weaken the structure of the airway, which leads to expiratory collapse. In adults, trauma to the tracheal cartilage from prolonged tracheostomy tube placement or recurrent intubation is the most common cause of acquired tracheomalacia.^[2]^ The diagnostic criteria for tracheomalacia have not been clearly established. However, evaluation of the cross-sectional area of the trachea with dynamic computerized tomography and the narrowing of the airway lumen (>50%) during expiration with flexible bronchoscopy can be highly accurate in identifying tracheomalacia.^[11]^

The structural weakness of tracheal cartilage owing to tracheostomy and indwelling endotracheal tubes is closely related to the inflammatory process. Prolonged placement of a tracheostomy tube and elevated cuff pressure lead to airway irritation, increased secretions, and ischemia.^[3]^ In addition, frequent exposure to positive pressure ventilation can exacerbate airway damage by causing tracheal overdistention, decreased tracheal smooth muscle thickness and epithelial injury.^[12]^ These conditions cause acute or chronic tracheal inflammation (i.e., chondritis), which results in the vulnerability of the tracheal wall to injury by repeated contact with the distal end of the tracheostomy tube.

Bleeding at the tracheostomy site, regardless of its amount, can be life-threatening because it can lead to airway obstruction and aspiration. Continuously elevated pressure of the tracheostomy tube cuff in tracheomalacia patients may accelerate the weakening of the tracheal wall. This results in necrosis of the tracheal wall, erosion, and movement of the tracheostomy tube into adjacent blood vessels, including the innominate artery.^[13–15]^ The formation of a fistula between the trachea and innominate artery, also known as TIF, is a medical emergency in tracheomalacia patients with a high mortality rate. Although some patients with TIF present symptoms such as sentinel bleeding or hemoptysis prior to life-threatening hemorrhage,^[14,16–18]^ these cannot be used as diagnostic criteria or predictors of TIF. Sudden massive tracheal hemorrhage and blood aspiration make anesthetic management challenging. Emergency control of hemorrhaging plays an important role in the survival of TIF patients. However, immediate surgical ligation or arterial embolization is not easy due to poor surgical field visibility during massive bleeding. Aggressive volume resuscitation and prevention of blood aspiration are essential in anesthetic management.

In our case, surgical exploration caused injury to the weakened tracheal wall and innominate artery of the patient, which resulted in the formation of a fistula. As this condition was unpredictable, it was fortunate that we removed the tracheostomy tube and secured the patient’s airway with an endotracheal tube before massive bleeding occurred. Additionally, as soon as massive hemorrhaging began, we immediately placed the tip of the endotracheal tube at the bleeding site by checking the bronchoscope and hyperinflated the cuff. This compression with the overinflated cuff was very effective in reducing bleeding and preventing the aspiration of blood, which was helpful in stabilizing vital signs and minimizing postoperative complications. Compression of the innominate artery with a rigid bronchoscope^[3]^ or finger pressure^[19,20]^ (Utley maneuver) is generally recommended, but we could reduce bleeding significantly only with overinflation of the endotracheal tube cuff. As compression with the cuff optimized the visualization of the surgical field, the surgeon could easily locate and access the injury. It was meaningful that anesthetic management could decrease the duration of surgery and improve surgical outcomes.

Although our handling of the emergency situation was successful, the outcome of the management in tracheomalacia patients with active bleeding due to TIF is influenced by several factors. First, removal of tracheostomy and insertion of an endotracheal tube before active bleeding are essential to successful management. To inflate the cuff at the exact bleeding site as soon as possible by checking the bronchoscope, the insertion of the endotracheal tube must be performed. Second, the size of the inserted endotracheal tube is closely related to reducing bleeding and preventing blood aspiration. The compression effect with an overinflated cuff is more pronounced in larger endotracheal tubes than that in smaller tubes. Finally, the size and extent of innominate artery injury are also important factors in successful management. If the injury of the innominate artery is significant, the compression effect of reducing bleeding is very limited, so direct digital compression against the sternum of the innominate artery should be considered immediately.^[17]^ Most importantly, the possibility of TIF due to innominate artery injury should always be considered in tracheomalacia patients at any time during surgery. We recommend exchanging the tracheostomy tube for the reinforced endotracheal tube when anesthetic induction is performed in cases of surgery under general anesthesia in patients with tracheomalacia. Anesthesiologists should always monitor endotracheal bleeding and be prepared to use bronchoscopy.

In conclusion, innominate artery injury is a life-threatening complication of prolonged tracheostomy in tracheomalacia patients. Immediate and appropriate overinflation of the endotracheal tube cuff by an anesthesiologist may provide improved surgical field visibility and time for critical surgical procedures in cases of massive hemorrhaging.

## Author contributions

**Conceptualization:** Yoo Jung Park, Hong Soo Jung.

**Data curation:** Yoo Jung Park, Eunji Kim.

**Writing - original draft:** Hong Soo Jung.

**Writing—review & editing:** Hong Soo Jung.
